# Autophagy in Idiopathic Pulmonary Fibrosis

**DOI:** 10.1371/journal.pone.0041394

**Published:** 2012-07-18

**Authors:** Avignat S. Patel, Ling Lin, Alexander Geyer, Jeffrey A. Haspel, Chang Hyeok An, Jiaofei Cao, Ivan O. Rosas, Danielle Morse

**Affiliations:** 1 Division of Pulmonary and Critical Care Medicine, Brigham and Women's Hospital and Harvard Medical School, Boston, Massachusetts, United States of America; 2 Department of Medicine, Pennsylvania State University, Hershey, Pennsylvainia, United States of America; 3 Division of Pulmonary, Critical Care, and Sleep Medicine, Mt. Sinai School of Medicine, New York, United States of America; 4 Lovelace Respiratory Research Institute, Albuquerque, New Mexico, United States of America; Helmholtz Zentrum München/Ludwig-Maximilians-University Munich, Germany

## Abstract

**Background:**

Autophagy is a basic cellular homeostatic process important to cell fate decisions under conditions of stress. Dysregulation of autophagy impacts numerous human diseases including cancer and chronic obstructive lung disease. This study investigates the role of autophagy in idiopathic pulmonary fibrosis.

**Methods:**

Human lung tissues from patients with IPF were analyzed for autophagy markers and modulating proteins using western blotting, confocal microscopy and transmission electron microscopy. To study the effects of TGF-β_1_ on autophagy, human lung fibroblasts were monitored by fluorescence microscopy and western blotting. *In vivo* experiments were done using the bleomycin-induced fibrosis mouse model.

**Results:**

Lung tissues from IPF patients demonstrate evidence of decreased autophagic activity as assessed by LC3, p62 protein expression and immunofluorescence, and numbers of autophagosomes. TGF-β_1_ inhibits autophagy in fibroblasts *in vitro* at least in part via activation of mTORC1; expression of TIGAR is also increased in response to TGF-β_1_. In the bleomycin model of pulmonary fibrosis, rapamycin treatment is antifibrotic, and rapamycin also decreases expression of á-smooth muscle actin and fibronectin by fibroblasts *in vitro*. Inhibition of key regulators of autophagy, LC3 and beclin-1, leads to the opposite effect on fibroblast expression of á-smooth muscle actin and fibronectin.

**Conclusion:**

Autophagy is not induced in pulmonary fibrosis despite activation of pathways known to promote autophagy. Impairment of autophagy by TGF-β_1_ may represent a mechanism for the promotion of fibrogenesis in IPF.

## Introduction

Autophagy is a catabolic process by which components of the cytoplasm are degraded in lysosomes. During this process, cytoplasmic cargo is sequestered within double-membraned autophagosomes and then degraded after fusion of autophagosomes with lysosomes. Although this morphological process was originally described half a century ago [1], the molecular mechanisms of autophagy have been only lately described, leading to recent interest in the role of autophagy in disease. Autophagy was first understood as a process by which organelles and macromolecules could be catabolized to remove damaged structures or to provide energy under conditions of nutrient starvation. The importance of autophagy to homeostasis and development has been underscored by numerous studies in yeast and mice [2], [3], [4], [5]. The function of autophagy in human disease and pre-clinical models of disease appears to be highly pleiotropic, however, and it is not always possible to predict the outcome of interventions that affect autophagy. In some instances, autophagy has been shown to be an adaptive pro-survival response, whereas under other circumstances autophagy appears to promote cell death and morbidity [6], [7]. Relatively few studies have addressed the role of autophagy in the lung; hence the role of autophagy in adult lung disease remains largely undefined. Autophagy in chronic obstructive lung disease (COPD) is greatly enhanced in the epithelium and appears to precede apoptosis and contribute to progression of disease [8], [9]. In alveolar macrophages of COPD patients, however, there is an impairment in autophagy [10]. In cystic fibrosis, there is evidence that mutant CFTR protein impairs autophagosome formation via depletion of beclin-1 [11]. Recently, it was demonstrated that tumorigenesis in tuberous sclerosis complex (TSC) which includes pulmonary lymphangioleiomyomatosis (LAM) is autophagy dependent [12], and Mi et.al. found that IL-17 antagonism induces autophagy and is protective in a mouse model of fibrosis [13]. Whether autophagy influences the outcome of idiopathic pulmonary fibrosis (IPF), a disease involving multiple cell types and pathogenic mechanisms, is unknown.

Recent advances in our understanding of the pathogenesis of IPF have underscored potential links with autophagy. Markers of ER stress have been shown to be increased in type II alveolar epithelial cells of IPF lungs compared with COPD or donor control lungs [14], and increased levels of ER stress promote fibrosis in experimental models [15]. ER stress is also known to induce autophagy [16], promoting cell survival [17] or death [18] in a context-specific manner. Markers of oxidative stress have also been identified in the lungs of IPF patients [19], and recent studies indicate that oxidative stress or elevated reactive oxygen species activate autophagy [20], [21]. Autophagy is also sensitive to oxygen tension and the hypoxia-inducible factor (HIF)-1α has been implicated as a major regulator of autophagy under hypoxic conditions [22]. Levels of HIF-1α are elevated in pulmonary fibrosis [23] and may contribute to disease progression [24], [25]. These associations suggest that autophagy is likely to be induced in pulmonary fibrosis. In this study, we used human tissues, the murine bleomycin model and an *in vitro* transforming growth factor-β_1_ (TGF-β_1_) model to explore the relationship of autophagy to fibrosis.

## Results

### Autophagy is not increased in IPF

**Figure 1:**
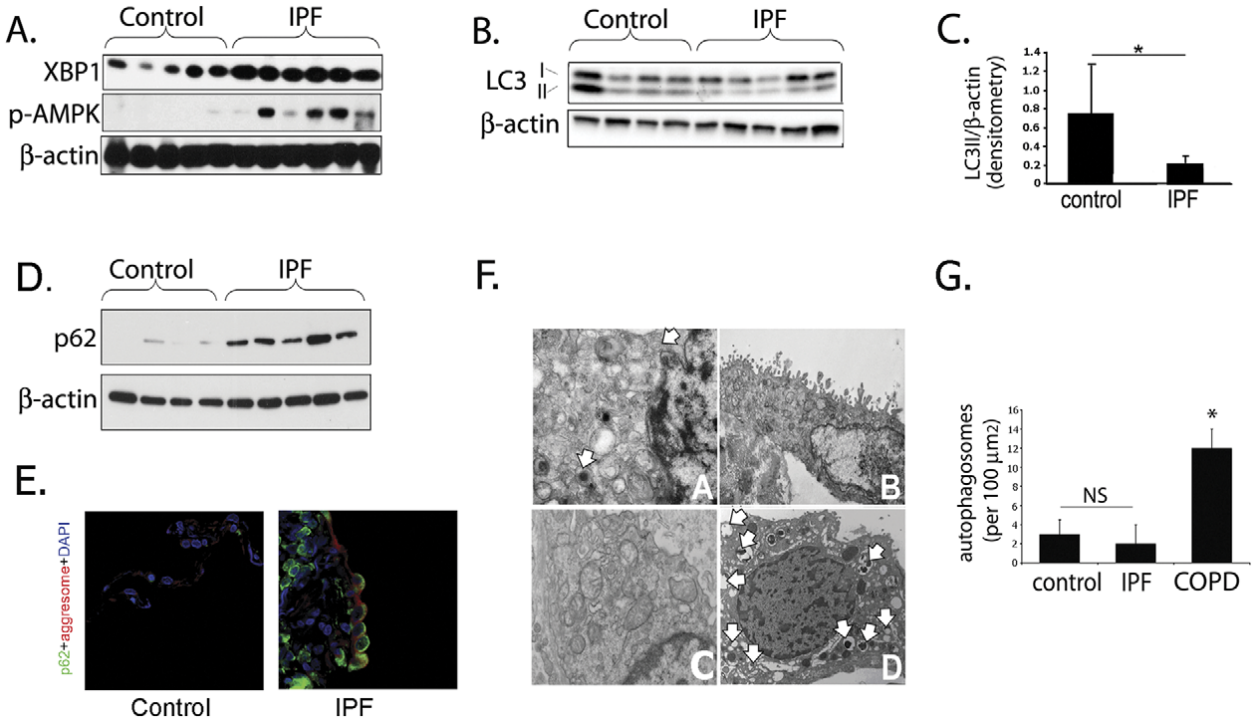
Autophagy is not increased in IPF. A) IPF whole lung homogenate demonstrates increased ER stress (elevated XBP1 expression) and increased phosphorylation of AMPK, factors which should drive autophagy. B) LC3-II (lower band) expression in IPF whole lung homogenate is decreased relative to control lung tissue C) Densitometry of Western blots demonstrating LC3-II level is lower in IPF than in control lung (*p = 0.05). D) Increased p62 in IPF lung suggests decreased autophagy. E) Immunofluorescence confocal microscopy of control and IPF lung tissue for p62 (green), aggresome (red), DAPI (blue) demonstrates increased p62 expression and aggresomes. F) Representative electron microscopy images from IPF (panels A, B), control (Panel C), and COPD (panel D); white arrows indicate autophagosomes. G) Quantitation of autophagic vacuoles in control, IPF, and COPD lung by EM demonstrates significantly higher numbers in COPD (*p<0.05 for IPF vs. COPD).

An immunoblot for X-box binding protein 1 (XBP1), a pivotal gene in the endoplasmic reticulum (ER) stress response, is shown in figure 1A. This immunoblot confirms previously published findings that ER stress is elevated in IPF lungs relative to control lungs [14], which would be expected to lead to increased levels of autophagy [16], [17]. Similarly, levels of phosphorylated AMPK (pAMPK) are higher in IPF lung than control lungs (figure 1A). AMPK activation is a well known trigger of autophagy [26] and is phosphorylated in situations where ATP synthesis is decreased (hypoxia, ischemia, low nutrient availability) or ATP consumption is increased. Despite evidence of ER stress and AMPK activation, we show in figure 1B and 1C that LC3-II levels are significantly lower in whole tissue homogenate of lungs from patients with IPF compared with control lungs from transplant donors without IPF. LC3, otherwise known as “microtubule-associated protein 1 light chain 3”, or MAP1LC3, is commonly used to monitor autophagy in cultured cells and animal tissue. The cytosolic form of LC3 (termed LC3-I) is seen as the upper band in the immunoblot, and the autophagosomal membrane lipidated form (termed LC3-II) makes up the lower band [27]. We also show in figure 1D that p62 protein levels are higher in IPF than in control lung tissue. Levels of p62 (a chaperone molecule that carries cargo to the autophagosome for selective degradation) inversely correlate with autophagic activity [28]. We also assessed p62 levels by immunofluorescence confocal microscopy (Fig. 1E) and found higher amounts of p62 in IPF tissue than control, confirming the Western blot findings. Electron microscopy (EM), the gold standard for identification of autophagosomes, supports the Western blot and immunofluorescence results. In contrast to lungs of patients with COPD, which have previously been shown to exhibit elevated levels of autophagy [8], lungs from patients with IPF demonstrate only rare autophagosomes. This is shown in figure 1F panel D, where numerous autophagosomes in lung from a patient with COPD are labeled with white arrows; in contrast, few or no autophagosomes are seen in IPF (panel A, B) and control (Panel C) samples. Quantitation of autophagosome numbers is shown in figure 1G. Taken together, these data indicate that autophagy is not induced in lungs of patients with IPF despite activation of pathways known to increase levels of tissue autophagy.

### TGF-β_1_ inhibits autophagy in vitro

**Figure 2:**
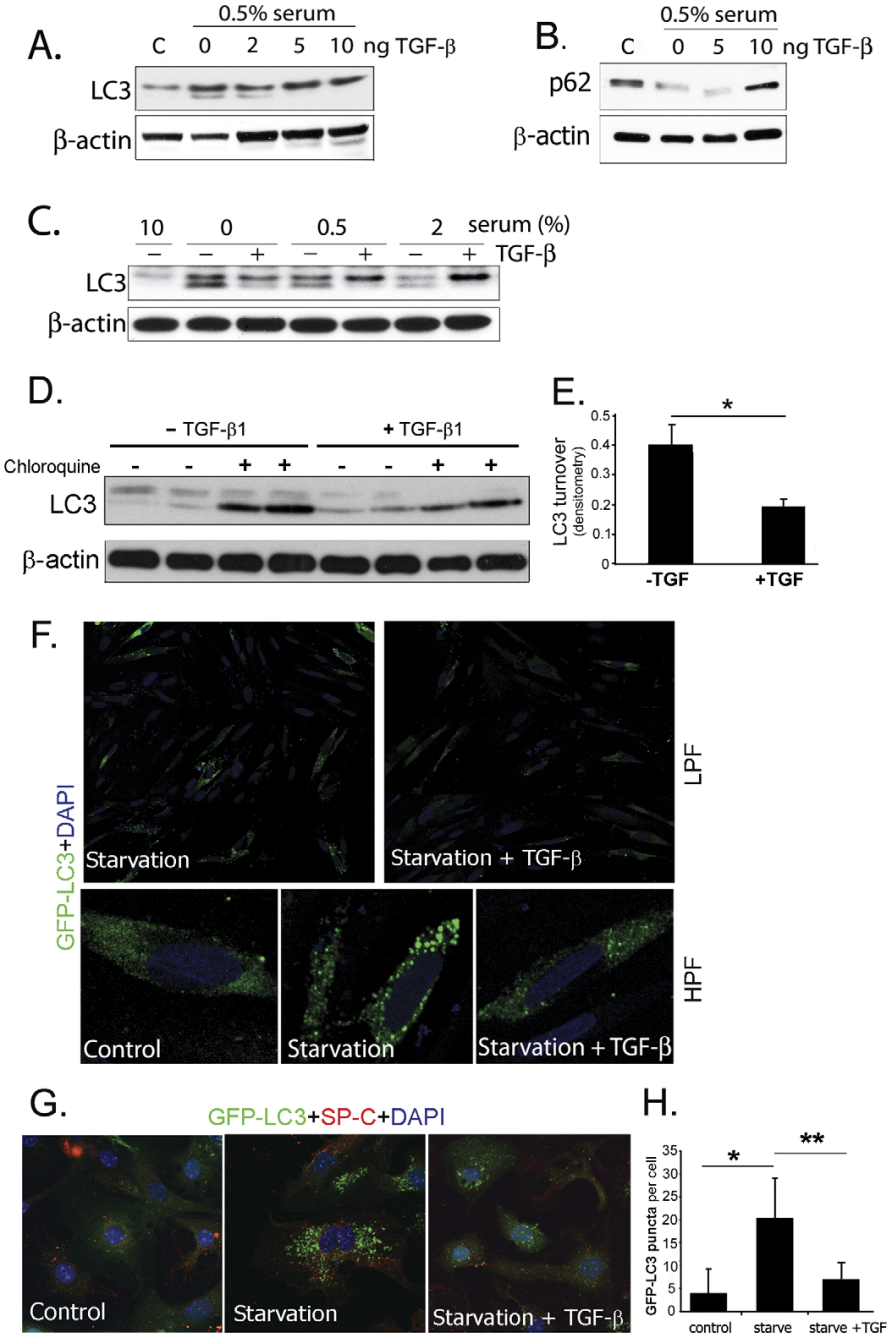
TGF-β_1_ inhibits autophagy *in vitro*. A) Human lung fibroblasts were cultured and treated with varying concentrations of TGF-β_1_. TGF-β_1_ inhibited activation of LC3, decreasing the intensity of the lower band on the western blot, and B) increased p62. C) Inhibition of LC3 activation by TGF-β_1_ with varying serum conditions. D) Fibroblasts treated with TGF-β_1_ for 24 hours and measurement of autophagic flux by LC3 western blotting (using lysosomal acidification inhibitor chloroquine). E) Densitometry of western blot shown in D (p = 0.045). F) Fluorescence microscopy of fibroblasts transfected with GFP-LC3 construct and stimulated with TGF-β_1_ showing that TGF-β_1_ inhibits formation of LC3 puncta. G) Confocal microscopy of type II alveolar epithelial cells isolated from GFP-LC3 transgenic mice and stimulated with TGF-β_1_ showing that TGF-β_1_ inhibits formation of LC3 puncta (green = GFP-LC3, red = SP-C, blue = DAPI). H) Quantification of GFP puncta per cell from figure 2G.

TGF-β_1_ is an essential mediator of fibrosis through its effects on fibroblasts [29], epithelial cells [30] and other cells of the lung. In order to test whether TGF-β_1_ would affect autophagy, we tested its effect on fibroblast cell lines *in vitro*. As shown in figure 2A, TGF-β_1_ inhibits conversion of LC3-I to LC3-II in a human lung fibroblast cell line in a dose-dependent manner in response to serum starvation. We also tested varying concentrations of serum from 0–2% and time points up to 48 hours and found the same effect (figure 2C), along with increased p62 levels implying reduced autophagy (figure 2B). To validate these conclusions we directly examined the effect of TGF-β_1_ on autophagic activity (or flux), as reflected by the turnover of LC3 in lysosomes [28]. For this experiment, fibroblasts were treated with 25 μM chloroquine or PBS 2 hours prior to harvest. The result was consistent with static LC3 levels, showing decreased flux (or LC3 turnover) in response to TGF-β_1_ (figure 2D, E). The images in figure 2F demonstrate the accumulation of punctate foci of green fluorescent protein (GFP) after starvation of fibroblasts transfected with a GFP-LC3 construct. The puncta are thought to represent autophagosomes [31], and therefore the paucity of puncta in the presence of TGF-β_1_ confirm a lower level of autophagy. Figure 2G represents GFP puncta formation in type II alveolar epithelial cells isolated from GFP-LC3 transgenic mice [27]. Similar to the effect seen in fibroblasts, TGF-β_1_ inhibits puncta formation in these epithelial cells confirming fewer autophagosomes (figure 2H).

### Inhibition of mTORC1 by rapamycin leads to improvement in bleomycin-induced experimental fibrosis

**Figure 3:**
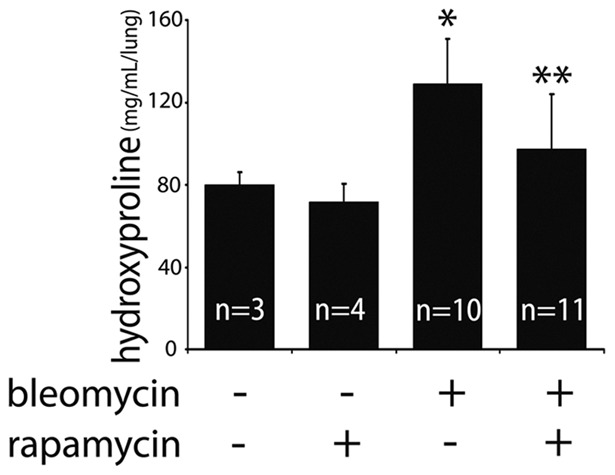
Effects of rapamycin on bleomycin induced fibrosis and autophagy. Hydroxyproline assay measuring lung collagen content demonstrates co-administration of rapamycin and bleomycin protects against fibrosis (*p = 0.003 for control vs. bleomycin; **p = 0.008 for bleomycin vs. rapamycin + bleomycin).

Since one of the primary modulators of autophagy is the mTORC1 pathway, we tested whether differences in the level of mTORC1 activity would affect the development of lung fibrosis in the murine bleomycin model by using the mTORC1 inhibitor rapamycin. We treated mice with oral rapamycin five days per week for 3 weeks following intratracheal bleomycin instillation. As shown in figure 3, mice receiving rapamycin in addition to bleomycin had significantly lower levels of lung hydroxyproline as a measure of collagen content than mice treated with bleomycin alone (97.3 vs 129.0 mcg/lung, p = 0.008).

### TGF-β_1_ activates mTORC1 and TIGAR

**Figure 4:**
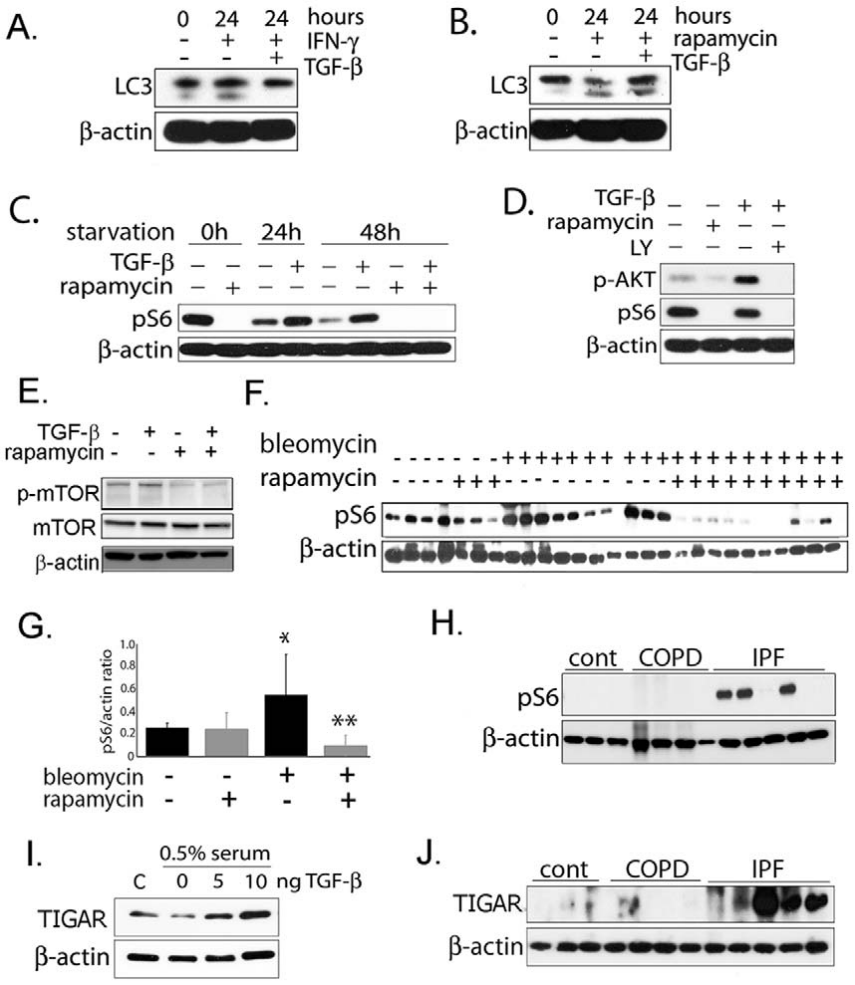
TGF-β_1_ activates mTOR and TIGAR. A) In human lung fibroblasts, TGF-β_1_ inhibits LC3-II formation, even in the presence of IFN-γ which induces autophagy. B) TGF-β_1_ is unable to inhibit LC3-II formation in presence of mTOR inhibitor rapamycin. C) TGF-β_1_ is able to activate mTORC1 which results in increased phospho-S6 but this activation does not occur in the presence of rapamycin. D) TGF-β_1_ appears to activate mTORC1 by activating upstream PI3K/AKT and treatment with PI3K inhibitor LY294002 prevents TGF-β_1_ induced mTOR activation. E) Western blot of phospho-mTOR (Ser2448) showing increased phospho-mTOR with TGF-β_1_ in fibroblasts and inhibition by rapamycin. F) Western blot of phospho-S6 from mouse lung tissue treated with bleomycin and rapamycin. G) Densitometry of blot from 4E. (*p = 0.03 for controls vs. bleomycin, **p = 0.003 for bleomycin vs rapamycin + bleomycin). H) phospho-S6 protein levels in human lung tissue is higher in IPF patients compared with COPD patients and healthy controls. I) TIGAR is induced by TGF-β_1_ in fibroblasts in a dose-responsive manner. J) Western blot demonstrating TIGAR protein levels in lung homogenate from human tissue is higher in IPF patients compared with COPD patients and healthy controls.

Since mTORC1 inhibition and autophagy induction in the murine bleomycin model decreased fibrosis and TGF-β_1_ decreased autophagy, we hypothesized that TGF-β_1_ might inhibit autophagy via activation of phosphatidylinositol-3-kinase (PI3K), Akt and mTORC1. Glucose-induced cell hypertrophy was also recently shown to involve TGF-β_1_ activation of the Akt-TOR pathway [32]. We therefore tested whether rapamycin is able to overcome TGF-β_1_ suppression of LC3 activation. As a negative control, we used interferon-γ to induce autophagy via a non-mTORC1 pathway. (Interferon is thought to induce autophagy via the immunity-related GTPase family M protein IRGM [33], [34]). As shown in figure 4A and B, TGF-β_1_ is capable of inhibiting autophagy in the presence of interferon-γ, but not in the presence of rapamycin. To explore this further, we examined the effect of TGF-β_1_ on phosphorylation of the S6 ribosomal protein. S6 ribosomal protein is phosphorylated by p70-S6 Kinase 1 (S6K1), which in turn is one of the most well-known targets of mTORC1. S6 phosphorylation is therefore commonly used to monitor mTORC1 activation [35]. As expected, rapamycin treatment resulted in inhibition of p-S6 (figure 4C). Starvation also led to a time-dependent decrease in p-S6 which was partially reversed by the addition of TGF-β_1_. Rapamycin fully inhibited the effect of TGF-β_1_ on S6 phosphorylation. The PI3K inhibitor LY-294002 abrogated Akt phosphorylation in response to TGF-β_1_ and also resulted in decreased ribosomal protein S6 phosphorylation (figure 4D). TGF-β_1_ also induces phosphorylation of mTOR at Ser2448 which has been theorized to correlate with mTOR activation (Figure 4E). To assess whether mTORC1 activity is increased in the setting of experimental lung fibrosis, mice were treated with intratracheal bleomycin alone or bleomycin and oral rapamycin 5 days per week. Lungs were assayed for S6 phosphorylation 3 weeks after initiation of treatment. As shown in figure 4F (and by densitometry in 4G), bleomycin treatment resulted in a significant increase in p-S6; as expected, this was abrogated by concomitant treatment with rapamycin. We then analyzed human lung tissue for p-S6 and found that on average, IPF patient samples have increased p-S6 expression compared to controls and COPD (figure 4H), but the phosphorylation is not uniform among the samples.

Another more recently described modulator of autophagy is TIGAR, or tp53-induced glycolysis and apoptosis regulator. The p53-inducible TIGAR protein functions as a fructose-2,6-bisphosphatase, promoting the pentose phosphate pathway. Expression of TIGAR has been shown to inhibit autophagy, via a mechanism thought to involve modulation of intracellular reactive oxygen species (ROS) [36], [37]. In figure 4I, we demonstrate that TGF-β_1_ is able to induce TIGAR expression under serum starvation in a dose-responsive manner. In addition, we examined TIGAR protein expression in human lung homogenate from control, COPD, and IPF patient samples (figure 4J). This shows that TIGAR is increased in IPF compared with controls and COPD. Therefore, TGF-β_1_ induction of TIGAR may be another mechanism by which autophagic activity is modulated.

### Inhibition of autophagy increases α-smooth muscle actin and fibronectin expression in fibroblasts

**Figure 5:**
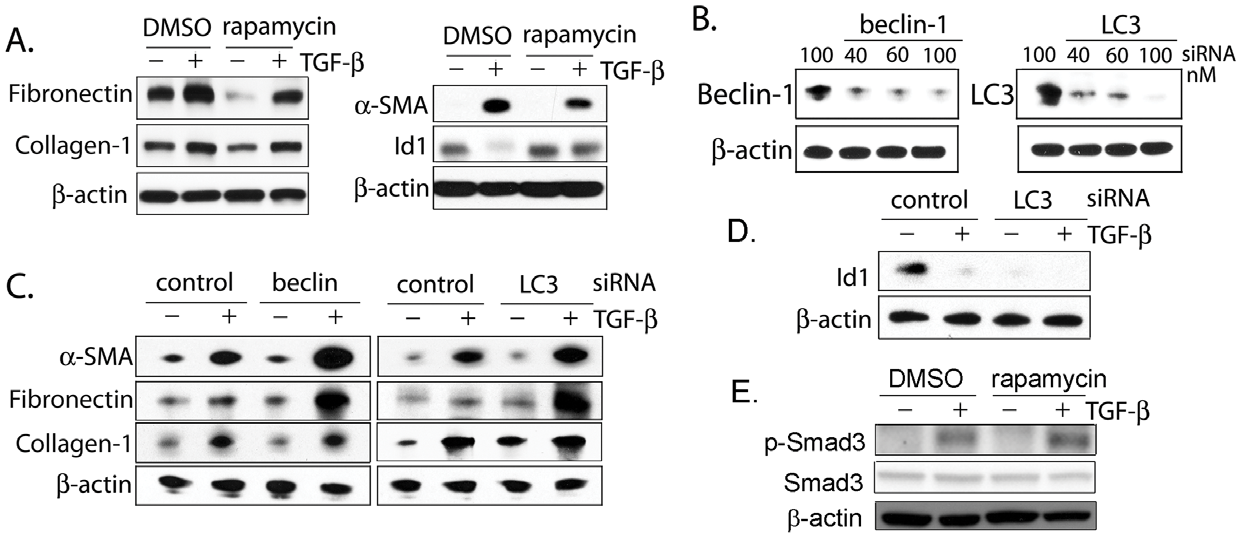
Inhibition of autophagy potentiates fibroblast activation. A) Rapamycin inhibits fibronectin and α-SMA expression in fibroblasts, and abrogates TGF-β_1_ suppression of Id-1. B) Beclin and LC3 siRNA effectively inhibit protein expression. C) α-SMA and fibronectin expression increases in fibroblasts with beclin and LC3 silencing whereas collagen is unchanged. D) When LC3 is silenced, Id-1 expression is lower than control. E) Rapamycin does not modulate phosphorylation of Smad3 in response to TGF-β_1_.

The effect of rapamycin on fibroblast matrix production has been examined by other investigators. Rapamycin does not appear to directly affect activation of Smad signaling or transcription of collagen-1; conflicting data exist as to whether rapamycin can inhibit collagen-1 production through post-translational mechanisms [38]–[39]. Rapamycin has been reported to slow anchorage-independent fibroblast growth and lessen TGF-β–mediated myofibroblast morphologic transformation and extracellular matrix synthesis [39], [40]. In keeping with these findings, we show in figure 5A that rapamycin treatment decreases fibroblast α-smooth muscle actin (α-SMA) and fibronectin expression in response to TGF-β_1_ and prevents suppression of the cytostatic inhibitor of differentiation-1 (Id1) [41] by TGF-β_1_. The expression of collagen-1 is unaffected by rapamycin treatment. We then tested whether inhibition of the progression of autophagy itself would affect fibroblast responses to TGF-β_1_. In order to inhibit autophagy, fibroblasts were treated with siRNA specific for either beclin (Becn1) or LC3 prior to TGF-β_1_ exposure (figure 5B). Interestingly, inhibition of either Becn1 or LC3 led to enhanced fibronectin and α -SMA expression in response to TGF-β_1_ (figure 5C). Expression of collagen-1 was unchanged by siRNA treatment. We also tested whether LC3 inhibition would affect levels of Id1, the absence of which has been shown to potentiate fibrosis [42]. As shown in figure 5D, Id1 expression was markedly lower in fibroblasts treated with LC3 siRNA than in control conditions. In Figure 5E, phosphorylated Smad3 was assessed by Western blotting on fibroblasts treated with TGF-β_1_ with and without rapamycin. This shows the expected induction of p-Smad3 with TGF-β_1_ but no change with rapamycin.

## Discussion

Our findings provide evidence that autophagy is not activated in the setting of IPF despite well described elevations in (ER) stress [14], [15], oxidative stress [19], and (HIF)-1α [23], which are all known to induce autophagy. We have shown that TGF-β_1_ inhibits autophagy in fibroblasts *in vitro*, and it is therefore possible that elevated TGF-β_1_ activity in the IPF lung accounts for this disconnect. Our studies using inhibitors of mTORC1 and PI3K suggest that the effect of TGF-β_1_ on autophagy is mediated at least in part via activation of PI3K, Akt and mTORC1, although the increased expression of TIGAR we observed may also play a role. In the bleomycin model of pulmonary fibrosis, administration of rapamycin, an inducer of autophagy [43], significantly reduced the degree of lung fibrosis. This is consistent with the findings of a previous study using a TGF-α overexpression model of pulmonary fibrosis; rapamycin was also shown to have a protective effect against fibrosis [44]. Other studies evaluating rapamycin in a rat bleomycin model and a murine systemic sclerosis model have discovered similar anti-fibrotic effects [45], [46], [47]. Finally, we have shown that inhibition of autophagic proteins LC3 and Beclin-1 leads to increased α-SMA and fibronectin expression by fibroblasts in response to TGF-β_1_, and that rapamycin has the opposite effect.

One of the main questions raised by this study is the mechanism by which autophagy modulates matrix and á-SMA expression. As autophagy represents a protein degradation pathway, it is reasonable to speculate that autophagy serves to degrade matrix molecules like fibronectin intracellularly before they are secreted. In a recent study by Arranguiz-Urroz, et.al.[48], the authors demonstrated that β_2_ adrenergic receptor agonism in cardiac fibroblasts activates autophagy leading to increased intracellular degradation of collagen. Another study demonstrated rapamycin decreases both fibronectin and collagen protein levels without affecting transcription [40]. More recently, Mi, et.al. showed that IL-17A antagonism protects against fibrosis by inducing autophagy and enhancing collagen degradation in epithelial cells [13]. In mouse mesangial cells, it has been shown that disruption of beclin-1 leads to collagen accumulation, both at baseline and with TGF-β_1_ stimulation [49]. In that study, induction of autophagy also led to intracellular collagen degradation. In contrast to our findings, however, TGF-β_1_ stimulated autophagy in mesangial cells, which may be due to differences in cell type and organ. Although we did not explore the dynamics of collagen turnover in the present study, our results do not indicate large differences in collagen-1 expression in response to LC3 and Beclin-1 inhibition or to treatment with rapamycin in lung fibroblasts. This is consistent with a previous study that also showed that rapamycin lessens TGF-β–mediated myofibroblast morphologic transformation without greatly affecting collagen production [39]. These findings, coupled with the fact that most extracellular matrix protein degradation does not occur intracellularly, indicate that this mechanism may not account entirely for the effect of autophagy on fibrosis.

Another potential explanation for the mechanism by which autophagy affects fibroblasts is a yet undescribed interaction between autophagy and TGF-β_1_ signaling pathways, either Smad-dependent or independent. Our data demonstrating that autophagy also affects Id-1 levels support this mechanism because Id1 is known to be both a target and modulator of Smad signaling [42] [41]. Despite this, we did not find a difference in phosphorylated Smad3 in cells treated with TGF-β_1_ and rapamycin. Thus, an alternative element of the Smad signaling pathway may be involved or a Smad-independent mechanism may account for the effects of autophagy on fibroblasts. The nature of any interaction between TGF-β_1_ signaling and autophagy will need to be further investigated in future studies.

The relationship between ER stress and IPF have been highlighted by a number of investigators [14], [15], and this represents another mechanism by which increased autophagy could be anti-fibrotic. Besides being potently induced by ER stress [50], autophagy is thought to play a vital role in clearing intracellular protein aggregates, thus modulating cell survival during the unfolded protein response [50]. While epithelial ER stress in IPF is thought to be due to misfolding of aberrant proteins rather than defective clearance [51], it may be that the lack of autophagy exacerbates this process. Increased accumulation of protein aggregates in the absence of autophagy could worsen ER stress and promote epithelial cell death.

The demonstration of opposite effects of rapamycin treatment and inhibition of LC3 or Beclin-1 suggests that rapamycin exerts its effects on fibroblasts by modulating autophagy but by no means proves this point. mTORC1 also has well-described key homeostatic functions as a regulator of protein and lipid biosynthesis, cell growth, and energy metabolism [52]. Inhibition of these essential functions of mTORC1 by rapamycin, apart from the drug's induction of autophagy, could affect fibroblast differentiation, matrix production and the development of fibrosis *in vivo*. This alternative mechanism could involve suppression of TGF-β_1_ signaling via mTORC1 or it could be completely independent of TGF-β_1_. There are inconsistencies in the literature with regard to the role of mTORC1 in TGF-β_1_ induced fibroblast responses. Goc, et.al. showed that ECM synthesis is inhibited in Akt1−/− fibroblasts and in wild-type fibroblasts treated with rapamycin [40]. In contrast, Rahimi, et.al. found that rapamycin did not alter fibronectin or collagen transcript or protein levels despite evidence that TGF-β_1_ activates mTORC1 resulting in increased proliferation [39]. These studies do not address whether any of rapamycin's effects can be specifically attributed to autophagy induction. The ability to induce autophagy in experimental systems via an mTORC1 independent pathway would help differentiate mTORC1's various functions, but this remains a challenge for the field. Although chemicals and drugs have been shown to induce autophagy independent of mTORC1, (e.g. amiodarone, clonidine, calcium channel antagonists), they often need to be combined with mTOR inhibitors to achieve significant modulation of autophagy [28]. The results of our experiments using LC3 and Beclin-1 silencing support the idea that specifically inhibiting autophagy (without changing mTORC1 activity) can alter fibroblast responses to TGF-β_1_. However, more definitive studies will be required to explore mTORC1 dependent and independent methods of activating autophagy and the effects they have on fibrogenesis. Future experiments examining mice with genetically deficient autophagy may help to address whether induction of autophagy is required for the antifibrotic effect of rapamycin.

Another limitation of our study is the use of serum-starvation for induction of autophagy *in vitro*. We acknowledge that induction of autophagy through serum starvation is not reflective of conditions in human disease. However, to demonstrate TGF-β_1_'s inhibitory effect we required the presence of a detectable level of basal autophagy. Therefore, despite its limitations, we used the most commonly employed method of accomplishing this. In addition, it is worth noting that a vast literature on TGF-β_1_ biological effects has been generated using serum-starved cell lines. It is interesting to consider whether artificially low mTORC1 activity and elevated levels of autophagy in “control” cells has impacted our interpretation of decades of *in vitro* TGF-β_1_ studies.

Investigations into the role of autophagy in lung diseases have been growing over the last several years, and there is now strong evidence of its impact in COPD and LAM pathogenesis [8], [10], [12]. In cystic fibrosis, there is evidence that mutant CFTR protein impairs autophagosome formation via depletion of beclin-1 [11]. Because mutant CFTR is also cleared by autophagosomes [53], this creates a cycle in which dysfunctional CFTR accumulates inside the cell. Our study begins another chapter in the emerging story of autophagy in human lung diseases by investigating its function in pulmonary fibrosis. Since there are existing drugs that induce autophagy, this line of investigation offers the possibility of translation to patient care.

In conclusion, this study demonstrates that autophagy is not induced in idiopathic pulmonary fibrosis despite the upregulation of several activators of autophagy. *In vitro* experiments demonstrate that the pro-fibrotic mediator, TGF-β_1_, is likely responsible for decreased autophagy. Treatment of animals with an autophagy-inducing agent partially protects against fibrosis. Lastly, we have demonstrated that inhibition of autophagy promotes extracellular matrix production by fibroblasts, providing one mechanism by which autophagy can impact fibrogenesis.

## Materials and Methods

### Ethics Statement

This study involves the analysis of human clinical samples. All human lung tissues were obtained from either lung transplantation explant tissues or from lung resection from thoracic surgical cases. Lung tissue procurement was completed under clinical protocols 99-H-0068 and/or 04-H-0211, which were approved by the National Heart, Lung, and Blood Institute Institutional Review Board. Informed written consent was obtained from all study participants. All animals were housed in accordance with guidelines from the American Association for Laboratory Animal Care. Protocols were approved with Institutional Animal Use and Care Committee of Brigham & Women's Hospital and Harvard Medical School (Protocol #04551). All experiments were carried out in a manner to minimize suffering.

### Human Subjects

A diagnosis of usual interstitial pneumonia (UIP) was confirmed by a lung pathologist in all IPF cases. These lung sections were processed immediately after being obtained from thoracic surgeons. The isolated lung tissues were snap frozen and stored at −80°C at the core facility at the Thoracic Center of Excellence (COE) in Anatomic Pathology at the University of Pittsburgh or the Pulmonary and Critical Care Branch of Division of Intramural Research at the National, Heart, Lung and Blood Institute in Bethesda, Maryland.

### Transmission Electron Microscopy and Fluorescence Microscopy

Lung tissue sections were fixed in formalin and embedded in paraffin. Tissues or cells were photographed using a JEOL JEM 1210 transmission electron microscope (JEOL, Peabody, MA) at 80 or 60 kV onto electron microscope film (ESTAR thick base; Kodak, Rochester, NY) and printed onto photographic paper. Immunofluorescence on human lung tissue sections was performed with p62 (Sigma)/AlexaFluor 647 (Invitrogen), ProteoStat Aggresome Detection Reagent (Enzo LifeSciences), Hoechst 33343 nuclear stain (Enzo LifeSciences) and images were taken and processed with Olympus FluoView FV1000 confocal microscope. To examine the distribution of GFP-LC3 in fibroblasts, cells were observed under a fluorescence microscope and digital images were acquired for analysis (SPOT, Diagnostic Instruments, Inc.). To examine GFP-LC3 in the type II alveolar epithelial cells isolated from mouse lung, images were taken and processed with Olympus FluoView FV1000 confocal microscope and GFP puncta were quantified using ImageJ.

### Cell Culture, small interfering RNA (siRNA), and GFP-LC3 transfections

Human lung fibroblast MRC-5 cells (ATCC, Manassas, VA) were maintained in DMEM (Invitrogen, Carslbad, CA) with 10% fetal bovine serum and 1x penicillin/streptomycin (Invitrogen). Cells were incubated at 37°C with 5% CO_2_. 12 hours prior to treatment, media was changed to low-serum (0.5% FBS). Cells were then treated with 5 ng/mL recombinant TGF-β_1_ (Sigma-Aldrich T7039, St. Louis, MO) for 24h unless otherwise shown. For certain experiments, the cells were also cotreated with interferon-γ, LY294002, or rapamycin (1 μM). In autophagic flux assays, the cells were additionally treated with 25 μM chloroquine (Sigma) for 2h prior to harvest. For siRNAs, LC3 and Beclin siRNAs (SantaCruz Biotechnology, Santa Cruz, CA) were transfected according to manufacturer's protocol. One to two mg of GFP-LC3 (rat) was transfected into 26105 cells using Lipofectamine 2000 according to the supplier's protocol (Invitrogen 11668). For isolation of type II alveolar epithelial cells, two 6–8 week old GFP-LC3 transgenic mice (RIKEN BioResource Center, RBRC00806, Japan) were sacrificed. Their lungs were digested in dispase followed by negative selection process to remove macrophages and fibroblasts. The remaining cells were cultured for 48hours prior to treatment with TGF-β_1_ for 24 hours. The cells were then fixed in 4% PFA and co-stained with SP-C (Santa Cruz Biotechnology) and DAPI.

### Animal Model

C57BL/6J mice (Jackson Laboratories, Bar Harbor, ME) were treated with 0.075U of intratracheal bleomycin sulfate (Hospira, Inc, Lake Forest, IL) on day 1. A subset of mice were also treated with 50 μg rapamycin (Rapamune solution, Wyeth Pharm., now Pfizer, NY, NY) by oral gavage 5 days per week beginning at day 0 until lung harvest. The mice were sacrificed and lungs harvested on Day 24 and snap frozen in liquid nitrogen followed by storage at −80°C.

### Hydroxyproline Assay

The left lung from each animal was dried in a Speed-Vac for 12h, followed by 12 hours hydrolysis in 6N HCL at 110°C. The hydroylizate was then separated from particulate matter by centrifugation and neutralized with equivolume 6N NaOH. 15μl of each sample was then incubated with 100μl Chloramine-T solution [0.564g chloramine-T (Sigma C-9887), 4ml n-propanol, 32mL Citrate/Acetate Buffer] at room temperature for 20 min, followed by incubation with 100μl Ehrlich's Solution [4.5g DMAB (Sigma D2004), 18.6 mL n-propanol, 7.8 mL perchloric acid] at 65°C for 10 min. OD 550 was read and hyrdroxyproline concentrations calculated from standard curve generated using known concentrations of trans-4- hydroxyl-L-proline (Sigma H5534).

### Western Blotting

Western blot analysis was performed using protein extracted (using RIPA buffer, protease and phosphatase inhibitor cocktail) from lung homogenate or cell lysate using 4 to 20% Tris-glycine SDS-PAGE gels (Bio-Rad, Hercules, CA). Primary antibodies used were phosphoS6, phospho-AKT (Ser473), mTOR, phospho-mTOR (Ser2448), Smad3, phospho-Smad3 (Cell Signaling Technology, Beverly, MA), LC3B, β-Actin, p62 (Sigma), α-SMA (SantaCruz Biotechnology), TIGAR (Abcam, Cambridge, MA). The secondary antibodies were a goat anti-rabbit or goat anti-mouse horseradish peroxidase-conjugated antibody (SantaCruz Biotechnology).

### Statistical analysis

Results were expressed as means ± SD from at least three independent experiments. Differences in measured variables between experimental and control group were assessed by using the Student's t test. Statistically significant difference was accepted at P<0.05.
